# Highest Priority Health and Health Care Concerns of Transgender and Nonbinary Individuals in a Southern State

**DOI:** 10.1089/trgh.2018.0003

**Published:** 2018-12-18

**Authors:** Sarah Alexandra Marshall, Mary Kathryn Allison, Mary Kathryn Stewart, Noel D. Thompson, Dani S. Archie

**Affiliations:** ^1^ Department of Health Behavior and Health Education, Fay W. Boozman College of Public Health, University of Arkansas for Medical Sciences, Little Rock, Arkansas.; ^2^ Department of Health Policy and Management, Fay W. Boozman College of Public Health, University of Arkansas for Medical Sciences, Little Rock, Arkansas.; ^3^ Arkansas Transgender Equality Coalition, Little Rock, Arkansas.

**Keywords:** access to care, health insurance, South, transgender health, transition care

## Abstract

**Purpose:** Transgender (trans) and nonbinary (NB) individuals experience a number of health and health care disparities when compared with cisgender individuals. While this has been reflected in nationwide surveys of trans/NB people in the United States, few studies capture the unique experiences of trans people living in the South, and fewer studies have collected qualitative data directly from trans/NB people. The purpose of this trans/NB-led initiative was to engage the trans/NB community in a southern state in defining their most pressing health and health care concerns and comparing those results with those reported by their cisgender allies, as well as national samples of trans individuals.

**Methods:** Participants (*n*=125), who were trans/NB individuals (77%) and their cisgender allies (23%) living in a southern state, completed a survey with open-ended response options and/or participated in trans-led summits.

**Results:** The top three health and health care concerns identified by participants, both trans/NB and cisgender allies, were insurance coverage for transition-related care, access to and availability of transition-related care, and education of health care providers about trans patients and issues.

**Conclusions:** The top concerns from trans/NB participants and cisgender allies reflect health and health care issues frequently reported by the trans/NB community nationwide. Having qualitative data from trans/NB individuals and their allies living in the South enhances our understanding of these commonly reported concerns. Future research, education, and health care practice initiatives should focus on the concerns identified by the trans/NB community.

## Introduction

The prevalence of health disparities faced by transgender (trans) individuals has been well documented in the literature. Survey research suggests that trans and nonbinary (NB) individuals, defined as those whose gender identity or expression differs from the culturally bound gender associated with the sex they were assigned at birth,^1–4^ are more likely to face economic hardships, such as unemployment, poverty, and homelessness,^3–5^ are more likely to face violence and harassment,^3,4^ and are more likely to be uninsured^3,4,6,7^ than the general population. Also, transgender individuals face a higher lifetime risk for suicide attempt, with the highest rates of suicide existing among trans individuals who have faced poverty, violence, and discrimination.^3,4,7,8^

Utilization of gender-affirming medical treatment, also known as transition-related care, may be a protective factor for health disparities among trans individuals, particularly trans women, especially in mental health and substance use.^9,10^ However, access to affirming care is an issue for many trans/NB individuals. One out of four transgender individuals that completed the 2015 U.S. Transgender Survey reported experiencing issues with health care coverage, including being denied coverage for transition care, hormones, transition-related surgeries, or other routine care because they were transgender.^4^ Participants also reported negative experiences while accessing health care, including harassment, assault, refusal of care, or having to teach providers about the appropriate health care needs of transgender patients.^4^ These issues are particularly problematic for those in the southern United States.^11,12^

Religiosity and social conservatism, which are associated with lower rates of acceptance of transgender individuals,^13^ are more prevalent in the South,^14,15^ including the state of Arkansas—the focus of our study—which is one of the most highly religious states in the nation.^15^ Indeed, Arkansas is one of only three states (Mississippi and Alabama) in the country targeted by the Human Rights Campaign with their “Project One America” initiative, which invests substantial resources long-term in these states to address the stigma and discrimination experienced by LGBT individuals in these three southern states.^16^ For example, in the 2017 legislative session of the Arkansas General Assembly, at least 14 anti-LGBT bills were filed addressing issues, such as marriage equality, bathroom access for trans individuals, indecent exposure, birth certificates, employment, and housing discrimination among other issues.^17^

The impact of these attitudes being more prevalent in the south is supported by regional stratification of national data that tell us that transgender individuals living in the South are more likely to have experienced harassment at school, more likely to have lost a job due to bias, and less likely to have health insurance coverage, when compared with transgender participants living in other regions of the United States.^3,4^ Additionally, research has shown state-level variation among trans individuals and their experiences with being denied health care with states in the southern and western regions of the United States being notably worse indicating these attitudes are also likely more prevalent among health care providers.^18^ Furthermore, there is a need to focus on the particular state in which our study was conducted, as other researchers have identified Arkansas as one of four states (i.e., Arkansas, Texas, Oklahoma, and Louisiana) having the highest rates of psychological distress among trans people in the United States.^19^

At the same time, overall, little research has been conducted on the health and health care experiences and perceptions of transgender individuals living in the South^20^ and even in the most recent national survey, trans people living in the south were underrepresented relative to the population as a whole.^3,4^ While roughly 38% of Americans live in the South, only 18% (1,120) of the U.S. National Transgender Discrimination Survey participants^3^ and 27% (7,599) of U.S. Transgender Survey participants^4^ lived in the South. Therefore, better representation in research among this population, known to be experiencing greater barriers to quality care and higher rates of health disparities, is needed to inform intervention design and implementation.

Community-based participatory research is an important approach to engaging underrepresented populations in research^21^ and yet we know of few such studies that have been conducted in the trans community, particularly in rural regions of the south. This study seeks to address this gap in the literature and documents results of an engagement process driven by transgender community members, to identify the health and health care concerns and priorities of trans/NB Arkansans.

## Methods

The purpose of this project was to engage the trans/NB community who resided in Arkansas in defining their most pressing health and health care concerns. In fact, the engagement of trans patients in identifying what their own priorities are has been identified as a gap in the research literature.^22^ Using the principles of community engagement,^23^ academic researchers partnered with members of the trans/NB community to form a Research Working Group (RWG). RWG members worked together to develop and advertise the project, to create a facilitation guide for trans/NB-led summits to engage other trans/NB community members across the state, to recruit participants, and to design and disseminate a survey that was used as part of the data collection process. In essence, this was a trans/NB-led initiative. (The partnership development process has been reported.^24^) The project collected data through a survey that was distributed through multiple methods, including online, in person, and at nine summits over the course of 9 months beginning in July 2015 and ending in April 2016.

A letter of determination was submitted to the University of Arkansas for Medical Science's Institutional Review Board (IRB) describing our project. Since the focus of the project was on community engagement and not a systematic investigation designed to develop or contribute to generalizable knowledge, the IRB determined that the project did not meet the definition of human subjects research and was exempt from full review.

### Participants

Participants completing the survey and/or participating in the summits were trans/NB individuals and their cisgender allies, who were partners, family members, health care providers, and other community members invited by the trans/NB participants, and all of them resided in a southern state. Participants were recruited online through Facebook and e-mail invitations, as well as in person and by word of mouth. Including both trans/NB individuals and cisgender allies in the participant sample offered the study team an opportunity to potentially compare responses between groups. Particularly in terms of assessing the health care concerns of the trans/NB community, the study team could compare responses about concerns based on the lived experiences of the trans/NB individuals with the perceived concerns reported by their cisgender allies.

### Data collection

RWG members created a short, 17-item survey to collect demographic information from trans/NB participants as well as identify their priority health and health care concerns. The survey was initially created to be used as a facilitation tool during the summits, but was also distributed electronically through SurveyMonkey. The purpose of these summits was to provide a safe space where community members could share their concerns and work together to prioritize prominent community issues. Having the summits and ensuring that trans/NB individuals were provided an honorarium to facilitate the summits was recommended by trans/NB members of the RWG. The nine summits were held in six towns and cities in different parts of the southern state. To have a rapport with the participants, local trans individuals were recruited and trained to facilitate the interactive process for the members of their community. The RWG developed a facilitator's guide and summit materials to help the facilitators conduct the 2- to 3-h interactive process to inform the participants of the purpose of the project and to collect information about priority health and health care concerns. The trans/NB participants and their cisgender allies were in separate spaces for the discussion and data collection to allow participants the freedom to openly express themselves and to reduce bias in the concerns reported on the survey. At the end of each summit, the trans/NB and cisgender ally groups were brought together to discuss similarities and differences in each groups' perceived health and health care concerns.

To better capture a more diverse and representative sample of tran/NB individuals, the survey was also distributed in other ways. An electronic link to the survey was distributed through the project's website, through a Facebook group for trans/NB community members, and through e-mail contacts so that participants could complete the survey online if they were unable to attend an in-person summit. Paper copies of the survey were also hand delivered to community members who could not attend a summit or could not access the online version. Therefore, RWG members manually entered data from completed paper copies of the survey into SurveyMonkey so that all data could be housed in the online database until it was downloaded and analyzed.

The first page of the survey was a study information sheet that introduced the project's purpose and indicated that completing the survey was voluntary and anonymous. To maintain anonymity, there was no signed consent. The information sheet also included this statement, “By completing the survey, your informed consent to participate is implied.” Participants marked a box next to a statement indicating they had read and understood the study information and wished to complete the survey before proceeding to the survey questions.

Demographic questions addressed age, race, ethnicity, county of residence, highest education level obtained, household income, sex assigned at birth, current gender identity, sexual orientation, whether they had health insurance coverage, and employment, partnership, military, and student status. All information was self-reported. Participants were then asked to complete the open-ended question: “Please list up to five transgender health or health care-related issues you are most concerned about and you would like this research group to focus on in order of their importance to you.”

### Quantitative analysis

Frequency analysis of the demographic variables and the most pressing health/health care concerns among all respondents, as well as frequency analysis to compare responses between cisgender allies and trans/NB participants, were performed. Logistic regression analysis was also performed using RStudio to look for associations between demographic variables and priority health/health care concerns among all participants and particularly among trans/NB participants, which was the population of most interest for this study. Logistic regression analysis was performed to isolate variables predicting an outcome of individuals ranking one of the top three concerns identified in a frequency analysis of the coded qualitative results: “insurance,” “access to care,” or “provider education.” Demographic variables (i.e., Race, Age, Transgender Identity, Health Insurance Status, Relationship Status, and Employment Status) were evaluated through Pearson's Chi-Square Test to determine the presence of an association.

Answers to three of the demographic variables were used to create the “trans/NB” category. The three questions used were: (1) Do you identify as Trans? (Yes/no); (2) What was your sex assigned at birth? (Female, male, intersex, intersex assigned female, intersex assigned male); (3) What is your current gender identity? For current gender identity, multiple options were listed, including an open response “other” option, and multiple responses to this question were allowed. Those categorized as trans were defined as those who self-identified as trans, those whose current gender identity differed from the sex they were assigned as birth, or those whose current gender identity was MTF/Trans woman or FTM/Trans man. To show the distribution of these trans participants by self-identified gender identity, we categorized those who only selected NB identities (e.g., genderqueer, genderfluid, gender nonconforming, NB, neutrois, polygender, agender/genderless) as nonbinary, but those who selected both a NB and a binary identity (e.g., trans man/man, trans woman/woman) were categorized under their binary identity. Participants categorized as trans and/or NB were only separated to determine the frequency of gender identities represented in the data. These two identity categories were combined for the remaining analyses to achieve a large-enough sample in each cell.

### Qualitative analysis

To thoroughly examine the data and to ensure trustworthiness—or the credibility and dependability of claims made based on sound methodology—this study used multiple-analyst triangulation.^25^ Three researchers worked together to perform conventional content analysis^26^ of the stated prioritized health/health care concerns among the trans/NB community. Two of the researchers (M.K.A. and M.K.S.) independently collected, compiled, and sorted the responses from all participants while noting which responses were from trans/NB participants and which responses were from cisgender allies. Common themes emerged from this preliminary, independent review of the responses. These initial themes were compared, and 10 themes were identified. One of the senior researchers (M.K.S.) created a codebook with definitions of the 10 identified themes. Using this code book, she coded the responses from all participants. Then, a third researcher (S.A.M.) independently coded all of the responses using the same code book and set of defined themes. The coding was compared between these two researchers (M.K.S. and S.A.M.). They discussed and deliberated over each of the codes until they reached consensus and agreed with the coding of all of the responses. One of the initial researchers (M.K.A.) reviewed and confirmed that the coding generated by this process was appropriate. Then, all of the coded responses were counted using SPSS software. This calculation revealed the three most commonly reported themes from all participants. The researchers then separated the common themes reported by trans/NB participants from those reported by cisgender allies for comparison.

## Results

### Participant demographics

A total of 125 individuals completed the survey, 77% of which identified as trans/NB and 23% of which identified as cisgender. Four out of five survey participants were white. Approximately 9 out of 10 participants reported that they were not of Hispanic, Latino, or Spanish origin. The largest group of participants was between the ages of 22 and 29 years (30%). Approximately half of all participants were under the age of 30. One out of four participants identified their sexual orientation as heterosexual, whereas 15% identified as gay or lesbian, 41% identified as bisexual, pansexual, or queer, and 9% identified as asexual. Seven out of 10 participants were currently employed, and 88% of participants reported having health insurance. One out of four participants were currently enrolled as a student, and over half of participants reported a college degree. Roughly half of participants made less than $35,000 per year.

Among those that identified as trans/NB (77%), half were under the age of 30 and four out of five were white. Trans/NB participants were more likely to identify as bisexual, pansexual, or queer (46%) and asexual (12.5%) than cisgender participants (28% and 0%, respectively). When compared with cisgender participants, trans/NB participants were less likely to report having a college degree. A high school diploma or equivalent was the highest educational degree for half of the trans/NB participants, compared with 20% of cisgender participants. Only 10% of trans/NB participants had a graduate degree. Over 62% of trans/NB participants made less than $35,000 per year. Approximately 86% of trans/NB participants reported having health insurance, compared with 93% of cisgender participants. Trans/NB participants were also more likely to be single than cisgender participants. Over half of trans/NB participants were currently single, separated, divorced, or widowed, and 45% were partnered or married. Nearly 15% of trans/NB participants were veterans or active military. Refer to Table 1 for demographic information.

**Table 1.** Demographic Variables Based on Gender Identity of Participants (*n*=125)

	Trans/NB participants, *n* (%)	Cisgender participants, *n* (%)	All participants, *n* (%)
Total	96 (100)	29 (100)	125 (100)
Gender identity			
Man/trans man	30 (31.25)	12 (41.38)	42 (33.6)
Woman/trans woman	35 (36.46)	17 (58.62)	52 (41.6)
NB	31 (32.29)	N/A	31 (24.8)
Age			
13–21	20 (20.83)	2 (6.90)	22 (17.6)
22–29	32 (33.33)	6 (20.69)	38 (30.4)
30–39	17 (17.71)	3 (10.34)	20 (16.0)
40–64	23 (23.96)	11 (37.93)	34 (27.2)
65+	4 (4.17)	7 (24.14)	11 (8.8)
Race			
White	77 (80.21)	22 (75.86)	99 (79.2)
Racial minority	19 (19.79)	7 (24.14)	26 (20.8)
Hispanic, Latino, or Spanish origin			
Yes	8 (8.33)	2 (6.90)	10 (8.0)
No	88 (91.67)	27 (93.10)	115 (92.0)
Sexual orientation			
Heterosexual	19 (19.79)	14 (48.28)	33 (26.4)
Gay/lesbian	14 (14.58)	5 (17.24)	19 (15.2)
Bisexual/pansexual/queer	44 (45.83)	8 (27.59)	52 (41.6)
Asexual/other	12 (12.50)	—	12 (9.6)
Questioning/prefer not to say	7 (7.29)	2 (6.90)	9 (7.2)
Relationship status			
Single	43 (44.80)	5 (17.23)	48 (38.4)
Partnered	34 (35.42)	11 (37.93)	45 (36.0)
Married/civil union	9 (9.38)	11 (37.93)	20 (16.0)
Separated/divorced/widowed	10 (10.42)	2 (6.90)	12 (9.6)
Gross annual household income			
<$10,000	17 (17.71)	1 (3.45)	18 (14.4)
$10,001–$20,000	21 (21.88)	4 (13.79)	25 (20.0)
$20,001–$35,000	22 (22.92)	1 (3.45)	23 (18.4)
$35,001–$50,000	14 (14.58)	5 (17.24)	19 (15.2)
$50,001–$75,000	12 (12.50)	4 (13.79)	16 (12.8)
>$75,000	5 (5.21)	11 (37.93)	16 (12.8)
Unknown	6 (6.25)	3 (10.34)	9 (7.2)
Employment status			
Employed/self-employed	66 (68.74)	21 (72.41)	87 (69.6)
Unemployed	30 (31.25)	8 (27.59)	38 (30.4)
Have health insurance			
Yes	83 (86.46)	27 (93.10)	110 (88.0)
No	13 (13.54)	2 (6.90)	15 (12.0)
Highest educational degree			
High school or equivalent	48 (50.00)	6 (20.69)	54 (43.2)
Associate's degree	17 (17.71)	2 (6.90)	19 (15.2)
Bachelor's degree	21 (21.88)	7 (24.15)	28 (22.4)
Graduate degree	10 (10.42)	14 (48.28)	24 (19.2)
Currently enrolled student			
Yes	27 (28.13)	5 (17.24)	32 (25.6)
No	69 (71.88)	24 (82.76)	93 (74.4)
Veteran			
Yes	14 (14.58)	4 (13.79)	18 (14.4)
No	82 (85.42)	25 (86.21)	107 (85.6)

NB, nonbinary.

### Quantitative results

Based on frequency analysis after the qualitative responses were coded, the top three concerns among all participants were “insurance,” “access to care,” and “provider education.” Given the relatively small number of participants, statistical differences were hard to assess but a couple of associations are noteworthy. Regarding those who listed “insurance” as one of their top three priorities, the only statistically significant association found among the variables was with health insurance status. Those who reported having health insurance were significantly more likely to indicate “insurance” as a top health care concern (Chi-Square [*n*=83]=7.154, *p*=0.008). Additionally, individuals identifying as transgender were found to have an association with listing “provider education” as a top priority among those who listed this as one of their top-three most pressing concerns (Chi-square [*n*=62]=4.042, *p*=0.045).

### Qualitative results

The qualitative analysis revealed 10 themes that were of concern to the participants: (1) insurance coverage for transition-related care; (2) access to/availability of transition-related care; (3) education of health care providers about trans/NB patients and issues; (4) public education to address stigma and discrimination and non-health care systems change; (5) health care systems and policies that are supportive and trans/NB-inclusive; (6) access to trans/NB-knowledgeable mental health care providers; (7) concerns for transgender/NB/gender nonconforming youth; (8) physical health concerns; (9) suicide and suicide prevention; and (10) homelessness. These concerns represent a range of physical, mental, and social health-related issues identified by cisgender and trans/NB participants. Some of this information has been reported elsewhere.^24^

While a variety of concerns were reported by participants, the focus of this article is discussing the top three most frequently described health and health care-related concerns. These three concerns were each reported by 50% or more of both cisgender and trans/NB participants. Again, these are “insurance coverage for transition-related care,” “access to and/or availability of transition-related care,” and “education of health care providers about transgender patients and issues.” There was some difference in the order of priority of these concerns among all participants, but these three concerns were prominent in the responses collected from all participants, which is why they are given the most attention in reporting our results. Each of these issues are defined below and presented with illustrative quotes from the open-ended responses provided on the surveys.

### Theme 1: insurance coverage for transition-related care

This theme broadly included responses about “Trans-inclusive healthcare coverage across the board—federally, state, and employers” and specifically focused on insurance coverage for hormone therapy, gender-affirming surgery, laser treatment, and other care needed for gender affirmation. Many participants simply wrote responses like “insurance,” “lack of insurance,” “insurance coverage for trans-related surgery,” “coverage of hormones,” or the “cost of T.” A couple of participants even used phrases like “insurance discrimination.” This theme included responses about the cost of transition care as well as the denial of insurance coverage for typically covered care because of a person's transgender status (e.g., denial of coverage for cardiovascular disease-related costs based on an argument that the health issues experienced are due to a person's estrogen use). One trans participant said they were concerned about “why most insurance [companies] don't feel as if the [treatment] we need is a need when it truly is a need.” Cisgender participants also prioritized the need for comprehensive “health insurance covering mental health/hormone/surgery for transgender/nonconforming individuals.”

### Theme 2: access to/availability of transition-related care

Responses within this theme about “access” or “availability” of transition-related care centered around concerns of being able to find health care providers who will prescribe hormone therapy and/or gender-affirming surgery as well as access to quality care for needs associated with the sex assigned at birth, information on how to transition, and transportation to transition care providers. Since “access” could be defined in different ways, many issues related to access to care are encompassed within this theme. For instance, one trans participant said, “Accessibility to primary care physicians [who were] affirming and knowledgeable” was a concern, whereas another person wrote “availability of hormones” was a concern. Cisgender participants also prioritized “access to medical care for transition” and “availability of hormones for those who are interested.”

### Theme 3: education of health care providers about transgender patients and issues

When referring to “providers” within this theme of “provider education,” this term includes physicians, nurses, pharmacists, and other health care professionals and staff. This theme focused on the need for providers who are culturally competent to care for trans/NB patients, and included remarks such as, “Doctors and nursing and other staff being educated about being respectful to trans folks.” That is, providers who will not misgender or discriminate against patients for being trans but rather will treat trans/NB patients with respect. One person said it this way: “Having as much respect from healthcare professionals as cisgender people receive.” The main focus in this theme recognized a need for “informed” health care professionals—those that are knowledgeable about transgender issues and able to provide quality health care to this population for both trans and non-trans-related care to avoid experiences, such as “being treated like something is seriously wrong with me and my intelligence by medical personnel.” To address this concern, trans participants suggested “education among healthcare workers relating to how trans bodies and minds work” and “provider networking [and] education building.” One cisgender participant asked, “what type of training/curriculum is available to educate future providers on trans health issues?,” suggesting the need for education specific to trans health issues in medical training.

## Discussion

The top three health-related priorities among our participants, both trans/NB and cisgender allies, were insurance coverage for gender-affirming care, access to providers able and willing to provide such care, and provider education. Both our trans/NB and cisgender respondents had similar ways of describing these priorities, as illustrated by quotes presented in our results. Considering that the cisgender participants were mostly partners, family members, or close friends of the trans/NB participants, the similarity in their prioritized health and health care concerns may be due to the fact that these individuals are strongly engaged with the experiences and needs of their trans/NB partners, friends, and family members. These prioritized health and health care concerns take on greater weight in the conservative cultural context of this study, which was conducted in one of the most highly religious states in the United States.

Interestingly, one of the few significant associations in our regression analyses indicated that participants who reported having health insurance were more likely to list coverage for gender-affirming care as a top concern. This finding is not surprising given that most health plans in Arkansas explicitly exclude coverage for such care. While insurance coverage increased significantly among low-income Arkansans under the Affordable Care Act (ACA),^27^ coverage for transition-related care through marketplace health plans was not included at the time of our survey in 2015. While nondiscrimination mandates in Section 1557 of the ACA scheduled to go into effect in January 2017 were interpreted under the Obama administration as transgender inclusive, a nationwide preliminary injunction by federal Judge Reed O'Connor in Texas in December 2016 barred these mandates from being enforced.^28^ This lack of coverage creates significant access barriers for trans/NB patients seeking providers who are willing and able to offer gender-affirming services because lack of reimbursement is a known disincentive for providers, including those who are willing to provide such care.^29^

Echoing our findings, current literature confirms that transgender individuals report health care access issues, especially transition-related and gender-affirming care.^30^ Larger provider networks are needed to improve patient access to trans-competent care and to facilitate providers' referrals for specialty care. Both patients and health care providers can face challenges identifying health care professionals able to provide quality care for trans/NB patients. Studies show that providers struggle to identify and make referrals to other providers more competent to care for their transgender patients,^2,31^ which can be especially true in socioculturally conservative, rural states such as Arkansas. In a 2012 survey of the Liaison Committee on Medical Education, <9% of participating institutions had procedures to identify LGBT-competent physicians, and only 15% of participating institutions had an available list of LGBT-competent physicians affiliated with their institution.^32^ Without health care resource lists of trans-friendly providers, providers have to rely on personal contacts for patient referrals. Furthermore, while having a resource list of trans-competent providers for both physicians and patients is important, creating and maintaining such a list is not a simple task. In Arkansas, the Arkansas Transgender Equality Coalition (ArTEC) website includes a health care resource directory that lists transition care providers and gender-affirming providers in multiple specialties, but keeping the list up to date can be both challenging and time intensive, requiring ongoing feedback from trans/NB community members about their health care experiences statewide.

Survey participants also prioritized the need for provider education to improve access to culturally competent, “trans-friendly” providers who will treat trans/NB patients with respect by using their preferred name and pronouns and create a safe environment. While both cisgender and trans/NB survey participants prioritized insurance coverage and access to transition care, trans/NB participants were much more likely than their cisgender allies to list the need for provider education as a top priority, possibly reflecting their personal negative health care experiences. Previous research has shown that between 25%^4^ and 50%^3^ of transgender patients report having to teach their providers about transgender health. Those that had to teach their provider about transgender health were nearly four times as likely to delay care in the future.^33^ Other studies show that patients are more likely to delay care if they do not trust that their provider will act in their best interest.^2,34^ Also, another statewide survey of trans/NB adults found worse general health and greater odds of current depression and attempted suicide in the past year among respondents who reported delaying care due to fear of discrimination.^35^ Access to providers that are educated on transgender health and culturally competent language can increase a trans/NB patient's likelihood of seeking medically necessary care and lower their likelihood of preventable emergency department visits and hospital admissions.^2,36^ These studies highlight the importance of educating providers on transgender health issues and patient-centered care as a means to improve patient–provider interactions and thereby improve care-seeking behaviors and health outcomes among transgender patients.

In past studies, transgender patients have reported negative experiences with providers when accessing care, including mistreatment, verbal harassment, physical assault, denial of treatment, harsh language, and being blamed by their provider for their own health problems.^4,37–39^ These behaviors demonstrate providers' lack of cultural competence to appropriately and productively interact with transgender patients. Providers' failure to use patients' names or pronouns can lead transgender patients to feel that their provider is not affirming of their gender identity and may cause patients to question the ability of the provider to effectively render care. This suggests the need for trans/NB cultural competency training and education for health care providers to improve providers' behaviors and trans/NB patients' health care experiences.

In addition to lack of appropriate communication skills, studies show that health care providers often lack the knowledge and skills necessary to treat transgender patients for a range of health care issues^31,40^ and are unsure where to access information on transgender health.^40^ There is a lack of trans-specific health education and a gap in LGBT-related medical education in general throughout all levels of training: medical school, residency programs, fellowship training, and continuing medical education.^41^ A survey of medical schools in the United States and Canada found that the average time dedicated toward LGBT-related content in medical education was 5 h, and most medical schools reported no LGBT-specific instruction during clinical rotations in the third and fourth years when students are exposed to actual patients accessing care in a range of medical specialties.^42^

Previous studies have found that more than half of providers report lack of training in transgender-specific care and lack of exposure to transgender patients as barriers that interfered with their ability to provide appropriate care to transgender patients.^29^ Additionally, 71% of community pharmacy residents surveyed were not trained on transgender care and only 36% felt confident providing such care.^43^ Exposure to gender-confirming surgical training in the United States varies by specialty and regionally, with less in the south.^44^ Not only are students underexposed to transgender-related content in training and educational curricula in the health care professions, they also lack sufficient experience serving diverse sexual and gender minority patients, particularly transgender patients.^42^ Studies show that LGBT-inclusive medical curricula lead physicians to a better understanding of LGBT health issues and result in them taking more comprehensive medical histories of LGBT patients^7,45^; therefore, more training in providing LGBT-inclusive care—particularly transgender-specific care—is needed in medical and clinical curricula and should be integrated in medical school, residency programs, fellowship training, and continuing medical education.^29,46^

In addition to improving providers' ability to diagnose and treat transgender patients, provider education and training can influence providers' attitudes toward transgender patients.^7,38,46^ Interaction with real or standardized LGBT patients in medical school has been shown to increase the likelihood that physicians will have positive attitudes toward LGBT patients later in their careers.^7^ Additionally, offering three 2-h transgender health training sessions to medical staff was found to reduce negative attitudes toward transgender patients and improved transgender health-related clinical skills among clinicians at an urban medical clinic.^47^ Although research has shown that providing evidence-based information through medical education—which challenges the foundation upon which stigma is built—can reduce the prevalence of stigma among medical professionals, contact with the stigmatized group is the most effective strategy for reducing discriminatory behavior and stereotyping among providers.^38,48^ Implementing strategies to improve health care providers' attitudes toward transgender patients, in addition to improving knowledge and skills related to the care of these patients, are particularly important in states such as Arkansas with higher rates of religiosity and social conservatism.

Lack of training can also affect self-efficacy. Only 41% of endocrinologists surveyed felt “somewhat” or “very competent” to provide transgender care.^49^ Also, only 35% and 29% of obstetrics and gynecology (Ob/Gyn) providers surveyed nationally were comfortable caring for trans women and trans men, respectively.^50^ Providers' lack of knowledge of transgender-specific treatments and resources can also affect their ability to gather salient information about their patient's specific needs and to refer them for specialized care.^46^ Therefore, education and training is needed to improve health care providers' knowledge of trans/NB health and health care needs, attitudes toward trans/NB patients and their health concerns, self-efficacy to provide transgender care, and behaviors when treating trans/NB patients, including the use of affirming language and other communication skills.

In summary, our findings support current literature identifying barriers to care among trans patients.^51^ Members of the trans/NB community in a southern state have expressed their priority health and health care concerns, and even though this sample may not have been represented in previously conducted studies, their concerns reflect the issues reported. Having the qualitative data enhances our understanding of these commonly reported concerns, as well as informs the development and implementation of future education and research projects in this religiously conservative Southern state.

### Limitations

The researchers note a couple of limitations with this project. Namely a small sample size and having a fairly homogenous sample of mostly white respondents voicing their health and health care-related concerns may not seem like an accurate representation of concerns from the broader trans/NB community. However, many of these same concerns are also reported by larger and more diverse samples of trans/NB people.^3,4^ Also, due to small numbers, transgender and NB identities were lumped together, and the authors acknowledge that we did not explore the responses according to the variety of identities expressed by participants nor did we analyze the subgroups by sex assigned at birth. Perhaps with a larger sample, this study could be replicated and additional analysis by these subgroups would then be possible.

## Conclusion

Because the transgender community named health insurance coverage, access to transition-related care, and provider education as their top three priority health and health care-related concerns, future research, education, and health care practice initiatives should focus on these areas, especially those focused on the trans/NB community in the South. Transgender individuals have cited insurance coverage and the costs of out-of-pocket care as barriers to gender-affirming care in other research studies,^29,30^ but researchers should continue to investigate how lack of insurance coverage for transition-related care affects transgender individuals' health care experience and how the evolving national legislation regulating health insurance coverage impacts this unique population, particularly those living in conservative sociocultural contexts, such as the state of Arkansas.

The literature also confirms that transgender individuals across the country report issues with accessing transition-related care.^30,51^ This study enhances the literature by focusing on the voices of transgender and NB individuals from a southern state. Because this community prioritized access to transition-related care, future research should further investigate facilitators and barriers to transition care and the effectiveness of interventions to improve health care access. Additionally, researchers should investigate how and why the experiences of trans/NB individuals in the South might differ from those in other regions of the United States, as this would inform tailored interventions to improve health care access for trans/NB individuals in the South.

The participants of this study also prioritized provider education as a means to improve access to transition-related care and improve overall health care experiences for trans/NB individuals. The current literature has predominantly focused on investigating the lack of LGBT-related education among providers. Few studies have characterized the lack of transgender-specific clinical and cultural competency training and education among health care providers.^29,31,40^ Even fewer studies have characterized the lack of such training among health care providers in the South. Considering that education and cultural competency training have been shown to improve providers' attitudes and address biases,^7,38,47^ future studies should investigate both the level of trans-specific training among providers and the impact of such education on health care practice, patient experience, and health outcomes. Additionally, researchers should investigate how the knowledge, attitudes, and behaviors of health care providers in the South differ from those in other regions of the United States and tailor provider education and competency training accordingly.

## Abbreviations Used

ACA: Affordable Care Act
IRB: Institutional Review Board
NB: nonbinary
NIH: National Institutes of Health
NIMHD: National Center Institute on Minority Health and Health Disparities
PCORI: Patient-Centered Outcomes Research Institute
RWG: Research Working Group
